# Codifying Online Social Support for Breast Cancer Patients: Retrospective Qualitative Assessment

**DOI:** 10.2196/12880

**Published:** 2019-10-24

**Authors:** Jude P Mikal, Stuart W Grande, Michael J Beckstrand

**Affiliations:** 1 Division of Health Policy and Management School of Public Health University of Minnesota Minneapolis, MN United States; 2 College of Liberal Arts University of Minnesota Minneapolis, MN United States

**Keywords:** social support, social networking, social media, health communication, breast cancer

## Abstract

**Background:**

Social media has emerged as the epicenter for exchanging health-related information, resources, and emotional support. However, despite recognized benefits of social media for advancing health-promoting support exchange, researchers have struggled to differentiate between the different ways social support occurs and is expressed through social media.

**Objective:**

The objective of this study was to develop a fuller understanding of social support exchange by examining the ways in which breast cancer patients discuss their health needs and reach out for support on Facebook and to develop a coding schema that can be useful to other social media researchers.

**Methods:**

We conducted a retrospective qualitative assessment of text-based social support exchanges through Facebook among 30 breast cancer survivors. Facebook wall data were systematically scraped, organized, coded, and characterized by whether and which types of support were exchanged. Research questions focused on how often participants posted related to cancer, how often cancer patients reached out for support, and the relative frequency of informational, instrumental, or socioemotional support requests broadcast by patients on the site.

**Results:**

A novel ground-up coding schema applied to unwieldy Facebook data successfully identified social support exchange in two critical transitions in cancer treatment: diagnosis and transition off cancer therapy. Explanatory coding, design, and analysis processes led to a novel coding schema informed by 100,000 lines of data, an a priori literature review, and observed online social support exchanges. A final coding schema permits a compelling analysis of support exchange as a type of peer community, where members act proactively to buffer stress effects associated with negative health experiences. The coding schema framed operational definitions of what support meant and the forms each type of support could take in social media spaces.

**Conclusions:**

Given the importance of social media in social interaction, support exchange, and health promotion, our findings provide insight and clarity for researchers into the different forms informational, resource, and emotional support may take in Web-based social environments. Findings support broader continuity for evaluating computer-mediated support exchange.

## Introduction

### Background

More and more people are using social media to gather health information and access social support, and this trend has practical implications across health research and interventions. With nearly 7 in 10 adults using some type of social media, there are limitless opportunities for sharing, learning, and exchanging ideas [1,2]. Despite recognized benefits of social media for advancing lifestyle and wellness communication [3], researchers have had a much harder time differentiating between the different ways in which social support is expressed via social media. With some exceptions [4,5], research describing social support exchange via social media has struggled to provide a structure to the content and evolution of social media use following health and other transitions and trauma. Hampered by a tidal wave of not only social media data but also social media *platforms*, researchers have struggled to cast a net around a temporal swatch of data for a group of participants that is large enough to permit a systematic analysis of generalizable trends, as well as differences within and between subjects.

Researchers have used a number of research design and analysis techniques to permit a more systematic analysis of social media use corresponding to (or in response to) health transition and trauma. This includes data gathered from controlled or condition-specific sites [6-8], single snapshots of data or cross-sectional research design [9,10], or qualitative accounts of support exchange during times of health transition [11,12]. However, although each approach provides unique insights into the nature, quality, and health correlates of support exchanged via social media, the majority provide limited information on *how* social support is expressed or evolved in response to shifting support needs. Differentiating ways social support is given and received, what forms support takes on social media, and what online support means to users will advance learning beyond basic observation toward more robust analysis of this highly interactive environment.

At the outset, the goal of this study was to calculate the proportion of Facebook exchanges that included the exchange of social support. However, in codifying support exchange, a number of challenges emerged with respect to measurement and categorization of social support exchange on social media. In this paper, we present some of the unanticipated challenges associated with coding unwieldy social media data, the systematic approach our team used to manage those challenges, and the coding scheme that resulted. The goal of this paper is to lay the foundation for future studies coding, categorizing, and measuring social support exchanges via Facebook and begin to lay a foundation for analysis of how online support exchange comes to bear upon observed mental and physical health outcomes.

### Theoretical Framing

Social relationships and support have consistently emerged in research as important determinants of health (for a review, see [13,14]), but as the media through which those relationships are maintained grow and develop, so must the models used to describe them, their measure, and the techniques used to evaluate their effectiveness. In this review of relevant literature, we describe the evolution of social support research from the acknowledgment of a link between social networks and health, through the advent and widespread adoption of computer-mediated communication, to the persistent lack of clear and accessible published schemas for the systematic categorization of support in Web-based communities.

### Social Support and Health

As early as 1951, theorists have acknowledged the important stress buffering effects of social engagement [15]. Since then, scholars have demonstrated how participation in social organizations can create networks of support that diminish stress and ultimately bolster health [16-20]. Despite wide acknowledgment of the relationship between social relationships and health, the measurement and mechanisms through which support impacts health has been the subject of considerable discussion. For instance, whereas a study has proposed that support effectiveness is contingent upon structural aspects of the networks within which an individual is embedded [21], other study has focused on the stress buffering effects stemming from the nature and quality of the support exchanges themselves [21,22].

However, from this study, two notable continuities have emerged. The first is the distinction between the various types of support that can be exchanged. When support is exchanged, there is a general continuity in the research literature that such support will be informational, instrumental, or socioemotional. Informational support refers to information exchanged in response to an event or problem. Instrumental support refers to the exchange of resources designed to aid in coping, and socioemotional support refers to the support derived from feelings of togetherness, esteem, or belonging [23]. In addition, research has largely settled on three caveats for support effectiveness. In other words, to be effective, research has found that social support should be both empathetic [24] and responsive to the support needs expressed [22], and support should not appear burdensome to the support provider [25]. This presents important questions for support communicated via the internet that may diminish the cost of support exchange but simultaneously undermine key elements of support quality, such as fit and empathy.

### Social Support and Cancer

In addition to physical challenges, cancer diagnosis brings about a host of deleterious psychological impacts that last throughout treatment and into the transition to survivorship [26-28]. However, research shows that social support can buffer against the ill-effects of stress in three ways: (1) by bolstering morale and impacting threat assessment, (2) by impacting an individual’s behavior and promoting health positive behaviors, or (3) through biological processes, such as a lowered heart rate, altered hormone production, or improved immune function [13,14,29,30]. In the case of cancer patients, research has shown that strong networks of support can buffer against stress by bolstering mental and, by extension, physical health. And research shows that the implications of high or low social engagement are long-lasting. For example, research on breast cancer patients showed that lower levels of support at diagnosis were associated with a 4-fold increase in all-cause mortality and a 2-fold increase in mortality resulting from breast cancer [31]. And on the flip side, Salonen et al [32] found that received support had a positive impact on physical health and quality of life following breast cancer surgery. Even after the transition off cancer treatment, face-to-face networks of support have been shown to play an important role in determining physical health, post traumatic growth, and psychological well-being [33,34].

### Online Support-Seeking

Despite the known health benefits of positive face-to-face supportive interactions, cancer diagnosis and treatment may correspond to a time of limited support access. Patients may find that previously established networks of support are either unwilling to provide support or unable to meet the support needs that emerge throughout cancer treatment [5,35,36]. As a result, patients shift their attention to Web networks of support as possibly better suited to providing continuous access to consistent and fitting support [5]. With more than three-quarters of Americans accessing the internet between *daily* and *near constantly* [37], Web-based social relationships have become ubiquitous, and the internet has become an important tool in accessing not only health information but also dynamic networks of individuals coping with similar conditions. In addition, research shows that the internet is an effective medium for support exchange and offers a host of functional advantages over face-to-face support, including better fitting support [38], lower barriers to accessing support [39], increased control and privacy [40], and reduced reciprocal obligation [41].

### Internet-Mediated Social Support and Cancer

Research on support for breast cancer patients specifically has shown that computer-mediated communication is an effective medium for support transmission spanning from early diagnosis to treatment and into cancer survivorship. Early research on internet support groups for patients with breast cancer found that Web-based groups confer a host of mental and physical health benefits, including the following: improvement in quality of life, psychological symptoms, and coping response as well as reduction in pain [42-45]. Although trial evidence is minimal and limited, observational data strongly support the value of breast cancer support groups for quality of life and reduced feelings of depression and anxiety [7,46]. In a qualitative research study of 15 breast cancer patients, Hoybye et al [12] showed that social support communicated on the Web promoted personal empowerment and the exchange of knowledge between breast cancer patients and survivors. These results echo findings from a quantitative study of 206 breast cancer patients that showed that internet-mediated social support was associated with an increase in knowledge about cancer and its treatment options, along with a decrease in overall anxiety in patients [47].

However, with respect to Facebook—a multifaceted platform that provides access to varied networks of support—research has typically focused on support exchanged in contexts characterized by the same general structure: forum-style Facebook groups. Research results from this area of study have been mixed. Whereas a study shows that engagement with breast cancer support groups has the potential to promote empathy and the formation of personal relationships based on shared experiences [48], other studies suggest that the benefits of such groups can be exclusive, and the benefits of engagement are not distributed evenly among participants [49,50]. Nevertheless, the considerable variation within Facebook with respect to the media, and varied mechanisms of support exchange can make it difficult to differentiate types of behaviors observed on the Web and link those patterns with health benefits [1]. So, although Facebook groups are an important conduit of informational, esteem, companionship, or resource support during times of stress, they represent only one aspect of support exchange via Facebook. In this study, we aimed to create the building blocks to permit quantification, evaluation, and analysis of support exchange between individuals that could be used outside the context of Facebook groups.

## Methods

### Overview

The coding scheme developed by our team was designed to help organize unwieldy social media data, allow us to evaluate patterns in social media use, and measure changes in those patterns following transitions in cancer care. Methodical coding required a series of definitions and decisions based on previous literature, observational data, and bottom-up contextual analysis and those definitions are often edited out of papers and manuscripts because of space constraints. The purpose of this paper is to describe how the data created ambiguity in defining support and our rationale for decision making and operationalize and standardize the measurement of support communicated via social media, so that other researchers can adopt and adapt as needed.

To qualitatively assess the types of support exchanged through social media along with the challenges associated with categorization of these text-based interactions, we asked 30 breast cancer survivors to share their Facebook pages with our research team. Participants completed a short intake evaluation that included demographic information (sex, racial identity, language, family situation, and income), date of cancer diagnosis, date of transition off of cancer therapy, self-reported level of Facebook use (light, moderate, or heavy), and any observed changes in self-reported Facebook use following their cancer diagnosis or their transition off cancer therapy. In accordance with institutional review board approval, we asked participants to send a friend request to our research account to provide access to profile and page content.

### Inclusion and Exclusion Criteria

To be included in the study, participants had to have been diagnosed with stage 1, 2, or 3 breast cancer and out of treatment for at least three months. Participants had to have an active Facebook account for at least three months before diagnosis, though we did not specify a minimum threshold use. The Facebook profile needed to be set up three months before their initial date of diagnosis because we were interested in both diagnosis and transition off cancer therapy. We included only women who had been diagnosed with stage 1, 2, or 3 breast cancer and received chemotherapy treatment. To study both transitions, we did not include patients who had experienced a cancer relapse. Despite meeting all inclusion criteria, patients were ineligible for the study if they failed to complete the initial intake evaluation or were unable or unwilling to send our team a *friend request* on Facebook—as failure to do so prevented us from accessing the information on their Facebook walls.

### Participants

Our final sample comprised 30 women who had been diagnosed with breast cancer between 2010 and 2017. Our sample was exclusively white and female, and all 30 participants reported English as their primary language. The women in our study were aged between 32 and 63 years, with a mean age of 47 years. Overall, the majority of our participants (23/30) reported living with a partner or spouse. Of our 30 breast cancer survivors, 24 reported having at least one child. Study participants generally reported some college education (27/30). With regard to income, whereas 3 women refrained from reporting income, the remaining 27 participants were relatively evenly distributed between the 8 income categories listed in our intake evaluation. Median household income was between US $80,000 and US $99,999, and modal scores were split evenly between the US $60,000 to US $79,999 and ≥US $140,000 income categories. Demographic variables can be found in Table 1.

**Table 1:** Demographics.

Measure		Frequencies (n)
**Age (years)**		
	30-39	8
	40-49	10
	50-59	7
	≥60	5
**Partnership status**		
	Partnered	23
	Not partnered	7
**Number of children**		
	0	6
	1	4
	2	13
	3	5
	≥4	2
**Income (US $)**		
	Below 20,000	2
	20,000-39,999	2
	40,000-59,999	4
	60,000-79,999	5
	80,000-99,999	4
	100,000-119,999	3
	120,000-139,999	2
	≥140,000	5
	Prefer not to say	3

### Procedure and Coding

Capitalizing on Facebook’s unique timeline feature, we scrolled back to participants’ diagnosis date on their Facebook walls. To gather the Facebook data, we used a Google Chrome browser extension called Scraper that allows for specific sections of a viewed Web page to be copied directly into designated cells of a Google spreadsheet. The process involved navigating to the starting date of a given participant’s timeline, scrolling and allowing posts to load, and then activating the scraper extension to copy the loaded posts into a spreadsheet. We downloaded a total of 60 files: 2 for each of our 30 participants. One file included data for the 6 months centered on participants’ reported date of diagnosis, whereas the other for the 6 months surrounding participants’ transition off cancer therapy. Variables automatically downloaded included the following: participants’ and poster’s name; time, date, type (status update, picture, meme, or video), and the text of the post; and network repose characteristics such as number of likes, shares, and comments the post received.

### Analysis

In addition to the variables downloaded automatically by the Web-based scraping program, we were interested in quantifying support exchanges as a proportion of the total number of exchanges on participants’ Facebook walls. To evaluate that, our team conducted a line-by-line coding of all status updates and wall posts collected in our data scrape. Our coding team met initially to discuss social support definitions and how social support may be exchanged in social media environments. The coding team also shared academic articles and agreed on the original variables. Our early, theory-based coding scheme was coarse, including dichotomous variables for support exchanged and post valence, along with a categorical variable for type of support exchange.

In meeting 1, each member of the team was assigned a diagnosis and a termination file to review and code. We then met the subsequent week to discuss challenges in data coding and review any status updates or wall posts that could not be neatly categorized based on our 3-variable coding scheme. As challenges emerged with particular status updates, or more generally in observed trends and patterns, we revised our coding scheme and modified and recoded all previously coded data. We repeated this process weekly. Following the coding of our first 10 participants, we had settled on our final coding scheme that was used for the remaining 20 participants. However, our team continued to meet weekly to discuss any difficult-to-code or attribute status updates or wall posts. These were discussed with reference to our original research questions, and in all cases, we were able to reach a consensus.

Once all 30 participants’ data had been coded, each team member recoded 1000 lines of the data. These recoded status updates were then compared with the original codes to ensure high inter-rater reliability scores. Given the close collaboration of our team, our inter-rater reliability met all threshold requirement, with percent agreement between 75% and 95% across all variables and Maxwell random error scores between 0.74 and 0.93.

## Results

### Overview

Our first theoretically grounded coding scheme included all variables automatically downloaded by our browser extension, along with dichotomous variables for valence and support exchange and a categorical variable for type of support. Engagement with the data revealed a number of features of social media data that did not lend to neat theoretically grounded categorizations. The result of this engagement with the data was an iterative adaptation of established definitions of support to capture the variability inherent in Facebook data. In this section, we present our initial variables, challenges that arose from engagement with the data, and resulting modifications to our coding scheme. We present our variables in the order in which they appear in our database: post originator, post content, post valence, support provided, support requested, type of support, and response metrics, followed by a graphical depiction of our coding scheme and a presentation of the final database.

### Poster

Although approximately 79.34% (16,912/21,291) of top-level posts were written, posted, or shared by the participant, the remaining 20.56% (4379/21,291) were generated by Facebook friends as either posts made directly to the participant’s wall or updates, photos, or videos in which the participant was tagged. Participant-initiated posts were tallied separately from friend-initiated posts, and friend-initiated posts were used to assess the frequency of unsolicited social support interactions. Non–patient-initiated posts were not included in tallies of network response, which our team tallied using quantifiable measures of network response including the number of likes, comments, and unique commenters.

To evaluate changes in support-seeking behavior and network response, we restricted support transactions to those in which the patient was either receiving or requesting support. To contain support exchanges to those focused on the patient, all posts written by the patient in which support was transacted were coded as *support requests*. Posts written by a Facebook friend where support was transacted were coded as *support provision*. Tracking posts as patient- or friend-generated enabled us to exclude posts in which friends asked patients for support or where the patient provided advice, information, or resources to another member of their Facebook network. In addition, given that posts related to a patient’s own cancer could come from both a patient or a friend, using the originator of the post alongside the patient’s own cancer variable (discussed below) helped us distinguish self-disclosures (patient-generated or own cancer–related) from other discussions of the patient’s cancer (friend-generated or own cancer–related).

Distinguishing between patient- and friend-generated content may appear self-evident, but establishing authorship was not always clear. Patients’ Facebook pages often included content, memes, blog posts, and articles from other sources, and that content was often accompanied by an introduction or commentary generated by the patient. In an example, a patient posted a meme with a quote attributed to Kathy Kinney:

One day she finally grasped that unexpected things were always going to happen in life. And with that she realized the only control she had was how she chose to handle them. So she made the decision to survive using courage, humor and grace. She was the queen of her own life and the choice was hers.

Patient 23, aged 37 years, diagnosed in 2015

The meme was accompanied by a post by the patient relating to her own cancer:

This gave me strength as I started by new treatment today! It went so well that I left feeling way better than I’d started. Though that was likely due to the awesome [friend] and [friend] and their abundance of snacks…

Patient 23, aged 37 years, diagnosed in 2015

In this case, the content of the post originated in part from a second-party source, but the patient was responsible for the introductory text and stood behind the words as an expression of her own feelings with regard to coping and found relevance to her own experience with breast cancer and treatment. Another illustrative example comes from the husband of one of the breast cancer patients in our group. He logs onto the patient’s Facebook page to update the patient’s Facebook networks regarding her health status following surgery:

Staying overnight following [patient’s] surgery. She is doing well but as expected is in quite a bit of pain. Your prayers will surely help her through the night. “Like” this post to let her know that you are with us.

Patient 19, aged 48 years, diagnosed in 2013

These instances typify some of the problems faced in attributing content to individual users.

In these specific cases, the first example would have been attributed to the patient, whereas the second instance would have been eliminated from the analysis. These decisions were made with two research goals in mind. The first is that if the patient provides any text or advocates any sentiment be shared or expressed on her Facebook page, they can be seen as attributable to the patient. The second goal was to under-, rather than over-report, support exchanges through Facebook. Specifically, although the husband in the second example was disclosing information, and perhaps issuing a tacit request for support following surgery, the patient was not a part of the support exchange dyad, and thus, although there might be some residual benefit from the likes, responses, and comments, they were not in response to a direct request from the patient. In addition, through a different lens, the post from the husband could be seen as providing support to fellow friends and family who were concerned about the patient’s health status following surgery.

### Problem-Related, Cancer-Related, or Own Cancer–Related Post Content

Organizing cancer-related posts resulted in another important distinction within the category of cancer-related posts. We surmised early on that it was important to establish whether posts related to a problem or not. However, problem-related posts were not systematically cancer-related, and posts that were cancer-related were not systematically related to the patients’ own cancer. The goal of including a general *problem-related* variable rather than focusing exclusively on cancer-related posts reflected an objective from the outset to assess whether those who posted more often about problems before cancer diagnosis were more likely to post about cancer-related problems following diagnosis. In addition, patients often posted about non–cancer-related problems both before, during, and following the treatment. These posts discussed a gamut of issues both health-related and otherwise. For example, in the three months following breast cancer diagnosis, a patient posted:

Ugh. I got an IUD today and ladies who know, the pain suuuuucks...but I feel better knowing I’m ok to continue treatment with peace of mind. And I’m glad I didn’t have to pay for that! Ouchie :-(

Patient 36, aged 35 years, diagnosed in 2013

Another patient posted about job benefits, whereas a third posted a status update about the health problems her cat was experiencing:

Anybody experienced with kidney disease in cats? Our little Twinkie is not well. Poor sweetie. She’s only 4. This. Sucks.

Patient 14, aged 45 years, diagnosed in 2013

Distinguishing between problem-related, cancer-related, and own cancer–related posts will ultimately enable us to explore whether patients who post more about their problems before cancer diagnosis also post more about their problems following cancer diagnosis or other health-related trauma. In addition, by highlighting problem-related posts that did not related to cancer, we set the stage for an analysis of differential support availability following diagnosis. Specifically, do posts about cancer-related problems get more attention from Facebook friends than non–cancer-related problems?

An added complication in coding posts as either cancer- or non–cancer-related was that frequently *cancer-related* posts related to something other than the patients’ own cancer. Following cancer diagnosis, a number of participants became active in breast cancer campaigns—raising funds for breast cancer research and collecting supplies to send to such patients in the hospital. Breast cancer survivors in our study participated in both walks and races with fellow breast cancer survivors and made connections that ultimately impacted their Facebook network composition. As a result, several of the participants’ pages included pictures of fundraising activities, articles, or opportunities for friends and family to get involved either as potential donors or participants. Although these posts were cancer-related, and fundraising efforts were catalyzed by patient’s own diagnosis, the posts had very little to do with the patients’ own experience with cancer. We argue that the degree to which a post related to a patient’s own cancer experience would likely have implications for resulting support provided and, therefore, opted to include dichotomous variables for both *cancer-related* and *patient’s own cancer*.

All posts that related to the patient’s own cancer were coded as problem-related, cancer-related, and own cancer–related. This categorization allowed us to keep a register of the concerns and issues raised by the patients with respect to their own cancer, even when no support was requested. We labeled these posts as *self-disclosure* and interpreted them as tacit requests for support, in accordance with the study by Zhang et al [51]. This register of self-disclosures enabled us to index the types of issues discussed by the cancer patients. Self-disclosures could be general or specific and handled a range of issues from personal challenges to triumphs and updates on disease progression and cancer treatment. For example, a participant posted:

All 3 cousins got cancer before age 50. Unfortunately for two cousins their cancer was not detected early. Next week when I ride the Canary Challenge I will be celebrating my own “getting through cancer” as well as riding in memory of my special cousins...whose cancer was too far gone—sadly [cousin 1] passed at age 48 years, [cousin 2] died just a few months short of 53 years. I miss them both a lot.

Patient 24, aged 52 years, diagnosed in 2013

The post is quite general and focuses outward on others’ cancer experiences but, nevertheless, includes a brief mention of the participant’s own cancer experience as the motivator for participation in the Canary Challenge. Other self-disclosures were more specifically focused on the patient’s own experience with cancer diagnosis and treatment:

Hi all! I just went for my first walk around the ward. It went very well! Everyone here seems very happy and even impressed with my progress so far. I’ll be headed home this afternoon! ...thank you all for your overwhelming love, support, and encouragement!!! The docs say my lymph nodes are clear, so this chica is CANCER FREE!

Patient 26, aged 35 years, diagnosed in 2013

The second status update appears to serve a dual purpose of (1) updating friends and family on Facebook with regard to treatment progress and (2) inviting them to celebrate a treatment milestone, though the patient never explicitly requests support.

### Valence

We characterized all posts related to a patient’s own cancer as *problem-related* under the assumption that there was no positive development related to cancer (symptom abatement, treatment, or progression) that was not overshadowed by the problem of having the disease. In other words, rather than *good* and *bad* developments in cancer, the range could more reasonably be considered as varying between more and less bad. Evaluating valence, on the other hand, allowed us to distinguish between positive and negative developments in cancer and its progression. Patients could talk about developments in their cancer symptomology or treatment but put a more or less positive spin on them. For example, a patient provided updates on her progression through cancer treatment by including a countdown:

Health update! Radiation begins tonight. Only 7 more weeks to go!

Patient 23, aged 37 years, diagnosed in 2015

Another patient updated:

I heard from my doctor this week regarding the cancer gene- I do not have it. All tests came back normal so we can praise God for some good news. She did say because of the amount and location of lymph nodes involved they are considering me stage 3 so we are very thankful my treatments have started so soon and they are being so aggressive. Even though the nausea is not letting up, I’m remaining positive that the cancer is being killed. Thanks for the continued prayers and support.

Patient 38, aged 33 years, diagnosed in 2016

Whereas the valence in the first post is largely positive, that in the second post is more mixed. It includes both positive and negative developments in disease and treatment progression.

To decide on how to handle mixed-valence and other complicated posts, we went back to the research questions underpinning the inclusion of the variable in the first place. In this case, we opted to include a category to assess valence for two reasons. The first is that change in valence has been used to assess the quality of support exchanged in Web-based communities [52]. As a result, it is useful baseline information to have for any study that discusses the nature, quality, and particularly the effectiveness of online social support coded from Web-based social interactions. The second is that research has demonstrated a number of caveats to the positive relationship between online support exchange and improved health; and among other things, patients have cited forced positivity as the potential to diminish the quality, and by extension the health buffering effects, of supportive exchange. Thus, we were interested in whether Web-based social media systems, such as Facebook, were likely to reward patients whose cancer-related posts were characterized by more positive sentiment.

Despite a clear definition of valence, assessment was not straightforward. Some posts were characterized by clear valence. For example, a patient posted a *rant* about the unsolicited advice she received regarding self-care and cancer treatment:

Day 4 nicotine free! Saw my Mom’s fam tonight and told those who didn’t know about my current situation. It went well, no tears from me, and best of all no really upsetting “well, should you really do that…” TRUST me, this is moving fast for me too BUT it’s also my life, my choice. Please leave the second guessing to ME!!!! Rant: over.

Patient 36, aged 35 years, diagnosed in 2013

Posts of this nature, conveying a very clear valence, were comparatively rare. More typically, posts included a gamut of positive or negative emotions. Sometimes the positive and negative emotions were mixed together, as in the above quote. In other instances, posts began with a more negative or neutral disease progression update and ended with an expression of determination or gratitude. For example, a participant posted:

I am currently 1 week out from my second chemo treatment. I am 27 years old and have a 2 and 1 year old. You never know when it will be you. My mother always taught me to check and I found my triple negative by myself. Bless all those who have faught [sic] or who are fighting right along with me.

Patient 26, aged 32 years, diagnosed in 2012

The post begins with objective or possibly neutral information but ends with an expression of gratitude, first to the patient’s mother and then to other cancer patients. Guided by our notion of *forced positivity* in face-to-face interactions [36], we opted to interpret the inclusion of positive sentiment or gratitude as possible evidence of that patients felt compelled to shift the valence in their posts to receive a response from friends and family through Facebook. As such, status updates characterized by mixed valence were coded with respect to the final sentiment expressed in the post.

### Support Provided

Posts could only be coded as providing support if they were generated by a Facebook friend. Given that not all support exchanges that transpired between cancer patients and their support networks were support provided to the patient or support requests from patients, it was important to establish which support exchanges were most meaningful in the context of changes in support-seeking behaviors following breast cancer diagnosis. As such, we excluded any instances in which breast cancer patients were asked to provide support. In addition, coding top-level posts for evidence of support provision enabled us to establish the frequency of unsolicited support and measure it by support type: informational, resource, emotional, or general advice.

In our sample of 21,291 top-level posts coded, 5178 posts were unsolicited support posts from friends. As shown in Table 2, posts were roughly evenly divided between those around the time of cancer diagnosis (N=2457) and those surrounding transition off cancer therapies (N=2721), but there was a considerable 3-fold increase in unsolicited support provision immediately following cancer diagnosis.

**Table 2:** Code count and percentages of support requests.

Support request and types		Patient			Friend		
		Diagnosis (N=9322), n (%)	Termination (N=11,223), n (%)	Total (N=20,545), n (%)	Diagnosis (N=2457), n (%)	Termination (N=2721), n (%)	Total (N=5178), n (%)
Support requests		341 (3.66)	291 (2.59)	632 (3.08)	748 (30.44)	725 (26.64)	1474 (28.47)
**Support type**							
	Informational	53 (0.57)	36 (0.32)	89 (0.43)	22 (0.90)	27 (0.99)	49 (0.95)
	General advice	18 (0.19)	19 (0.17)	37 (0.18)	1 (0.04)	1 (0.00)	2 (0.04)
	Emotional	88 (0.94)	71 (0.63)	159 (0.77)	607 (24.70)	585 (21.50)	1192 (23.02)
	Resource	201 (2.16)	172 (1.53)	373 (1.82)	144 (5.86)	145 (5.33)	289 (5.58)

It is worth noting that among friend-generated wall posts, not all were intended to provide support. In addition, instances of support provision only accounted for 698 of the total 1329 total increase in posts between the three months preceding cancer diagnosis and the three months following it. This supports the notion that friends were generally checking in more with cancer patients following cancer diagnosis than they had been before diagnosis, though not always around cancer or expressions of social support.

Observations similar to the one above complicated the coding process with respect to support provision. To simplify, we operationalized support as exclusively those posts that contained encouragement, esteem support, information, resources, or the intention to transmit resources (for a full description of the definition of resource support, see the *support types* section below). Nevertheless, support provision was complicated in that it required coders to pay close attention to both macro- and microlevel contextual cues that could indicate whether a post was meant to provide emotional support and support was specifically cancer-related. In other words, in some instances, patients’ family members would post from their own account to provide information and rally support for the patient. The following example is from one of our participant’s daughters:

Tomorrow my mom will be having her surgery. Please continue to pray for her. These past six months have been difficult for her and our family and I appreciate all of the support everyone has given us. She has put up quite the fight and continues to keep strong. I love you, mom!

Daughter, patient 30, aged 63 years, diagnosed in 2013

This post provides encouragement and information on behalf of the patient and so despite not containing a direct message of support, it would have been coded as support provided. Conversely, friends often posted to participants’ walls general words of encouragement and support, such as *you rock!* Owing to the absence of indicators that the post was specifically meant to provide encouragement in the fight against cancer, we opted to estimate conservatively, calling the posts non–cancer-related transmissions of general emotional support.

Our sample also included several lengthy posts expressing support for cancer patients made to the friend’s own wall and simply tagging the cancer patient. On Facebook, when an individual creates a status update, they are able to tag a second Facebook user if that user is a friend. In so doing, the post will appear on both the original poster’s wall and that of any person tagged in the post. Our data showed a number of instances where a friend wrote a post about cancer and tagged a group of people that included the patient. One such instance came from a fellow breast cancer patient who tagged a group of 88 friends and posted:

Hopefully I tagged everyone! It’s been a hell of a year so far but I am very grateful for you all! I’m feeling so much better and ready for Chrissymas!!!

Friend, patient 25, aged 51 years, diagnosed in 2014

In these instances, it was unclear whether to interpret the post as providing support in that the original post was not directed at the patient herself. However, we opted to code the posts as both related to the patient’s own cancer and transmissions of actual support in that these were designed to express solidarity. It may have been an unwelcome expression of support or broadcasting of a particular patient’s health status, but the design of the post was to express support that was not directly in response to a request from the patient.

Microlevel context was also important but often quite difficult to assess. For example, a patient posted frequent chemotherapy countdowns. As a result, many of her network were aware of when her final chemotherapy was to occur and stopped by her page to offer words of encouragement. The words of encouragement never mention cancer specifically. For example, a participant posted a countdown to her final chemo treatment. As a result, on the day of her last treatment, she received numerous posts congratulating her—some referencing her final treatment, and some did not. One user wrote a note of congratulations and support but never mentioned cancer directly:

What a long hard ass journey and trail this has been. Not over yet, but the big hurdles are not behind us! We are blessed that we found the C when we did, for if not...things would not be the same right now. I have learned so much about myself, and strive to be the best husband I could be... Love you honey and congratulations!

Patient 16, aged 56 years, diagnosed in 2013

As in the above example, the nature and content of the post as well as surrounding posts made it clear that these were made in celebration of an impending completion of cancer treatment. In addition, in a number of instances, the support provided directly to a patient’s page by a friend was in response to a direct request for support from the patient. This type of support exchange occurring across top-level posts was quite rare. In addition, separating solicited and unsolicited support in top-level posts was beyond the scope of this study. As a result, we interpreted all friend-generated support-providing posts as examples of unsolicited support exchange.

### Support Requested

A common feature of research around Web- and non–Web-based support exchange is that support needs can be expressed both directly and indirectly. Indirectly, support needs may be expressed through the disclosure of personal information. Directly, individuals may ask for advice, information, or resources. As we had already parsed out problem-related posts from general cancer-related posts and posts related to a patient’s own cancer, our assessment was that we had captured the tacit support requests. As a result, we only coded status updates as *support requests* if the status update went beyond disclosing a need and actually issued a direct request for advice, encouragement, information, or resources. This greatly restricted the number of posts coded as support requests, but the decision was made to separate direct and indirect request in that such a separation enabled us to view differences in support availability on the basis of whether the post included a direct request for support or relied on more indirect methods of communicating a support need.

An additional complication that emerged in the coding of support requests was the requesting of support on behalf of a cancer patient. Posts were often posted directly to the friend’s wall but appeared on the patient’s wall because she had been tagged. Tagging the patient offers the benefit of ensuring wider broadcast; when a person is tagged in a post, the post is broadcast to not only the poster’s social network but also that of the patient. Although it was clear that the support requested was intended to benefit the breast cancer patient, it was unclear from these second-party support requests whether patients ever asked their friends to issue these calls for support. These posts differed from the general cancer condemnations in the *Support Provided* section above in that the posts made an express request for support on behalf of the cancer patient, rather than a simple condemnation of cancer. In the context of second-party posts, our team made the decision to count them as a provision of resource support in that they were allowing cancer patients to access support from a broader network of support.

### Type of Support

#### Typology Overview

If a post included a direct request for support or evidence of support being provided, we categorized the post by the type of support using established categories from social psychology literature [23]. Cancer patients are known to follow certain patterns in their support-seeking. Specifically, they tend to be selective about the source of various types of support—seeking emotional support from friends and family but preferring that informational support come from doctors or other breast cancer patients and survivors. Online support-seeking tends to follow a different pattern. Likely given the ease of transmission through the internet, research on computer-mediated communication suggests that, broadly speaking, users tend to gravitate toward emotional and informational support when exchanging support on the Web [36,53]. Beyond advancing research on the nature and quality of online support exchange, knowing the frequency with which various types of support exchanged could also help advance our understanding of cancer patients’ online support-seeking behavior. Specifically, do breast cancer patients behave more similar to other cancer patients in their support-seeking, or they seek the same types of support as other internet users?

To assess this, we began with a typology of three types of support as outlined in the theoretical framing of the paper. Posts that were assessed as either providing or requesting support were then evaluated for the type of support that was exchanged in the post. Following broad definitions laid out in the review of relevant literature above, we assessed whether each support exchange included transfer of information, resources, or socioemotional support. The categories are defined, and examples are provided in Table 3.

**Table 3:** Categories of provided and requested support, including definitions and examples from the data.

Support Type	Definition	Provided	Requested
I (Informational)	Transfer of relevant information to help cope with a problem	Hey lady…happy “last chemo” day! Came across a website…about all sorts of freebies, from wigs to housecleaning. Thought I would pass it along. (Patient 36, age 35, diagnosed 2012)	Calling all cooks – what’s your favorite Vegetarian/Vegan meals? I know there’s some of you out there. I am hoping to make more vegetarian meals in 2012. Stuff that’s kids like is a double plus! (Patient 13, age 41, diagnosed in 2011)
A (Advice)	General advice, not related to factual information	No instances of unsolicited general advice provided	Has anyone seen the new jungle book movie in the theater? Wondering if it’s too scary for [child]. He’s never been to a movie theater and I thought this movie might be a good one. (Patient 38, age 33, diagnosed in 2016)
E (Emotional)	Feeling of togetherness or the knowledge that one is valued	Miss [patient’s name]! How are you darling…it’s been a loooooonngggg time! Your little man is so adorable. Not sure what’s going on, but wanted you to know that I’m praying for you. Grab your strength from God…He provides us with all we need. Thanks for adding me! (friend, Patient 26, age 32, diagnosed in 2012)	Labs came back really low today, so if you’ve been sick or by someone sick please stay away. Say a prayer they go back up before my next treatment and that I get some energy. I’ve been exhausted the past two days, probably too much fun. (Patient 36, age 33, diagnosed in 2016)
R (Resource)	Actions and materials made available through individual support network	Consider helping this beautiful young mama, [tagged patient], if you can. Every little bit helps. Read her story. BREAST CANCER/ALL CANCER SUCKS!! (friend, Patient 29, age 35, diagnosed in 2016)	[Daughter]’s first day of school is tomorrow, and she’s very nervous…she’s coming in during the middle of the year. If you are her friend on fb or have her Snapchat…go flood her with encouraging messages please. Everyone else please say prayers for confidence, comfort, and nice kids to become good friends. Thanks! (Patient 29, age 36, diagnosed in 2016)

#### Emotional Support

Early on, our team made the decision to consolidate esteem support and social companionship under the auspices of emotional support. This is consistent with other coding schemes, as the distinction between esteem support (that one is loved or esteemed) and social companionship (that one is not alone) adds less in this context than a broad-level emotional buffering. Emotional support constituted the largest number of unsolicited support posts made by friends. In cases of support provision, the posts were very succinct and included statements, such as *you rock* or *you are so strong!* Longer message may be more specific to the person but include similarly general sentiment. For example, a friend of patient 17 posted the following to her wall:

Hey girilie [sic] Just wanted to stop by and say hi. I hope recovery is going great and hopefully alot [sic] less painful today then [sic] yesterday!!! Been praying for you:) How have you been otherwise, I see that you are blessed with two little ones they look so much like you:) Congrats on being a mama even though it’s a couple years late lol. Well hit me up whenever, I’m sure you’ll be online a bit more offten [sic] while you recover and I work in frunt [sic] of a computer so I am always on:) Have a great rest of you [sic] day and just know I’m praying for everything...fast recovery, little less pain eachday [sic], and yummy food made buy [sic] your honey for you:) Love yea Kim.

Friend, patient 17, aged 32 years, diagnosed in 2012

Despite the longer post content, the sentiment is quite similar to the shorter *you rock* type posts, offering general encouragement, a sense of togetherness, and small talk but limited engagement with the patient or their cancer.

In the context of support requests, emotional support was often requests for *thoughts*, *prayers*, *thoughts and prayers*, and even *positive vibes*, *mojo*, *karma*, or *luck*. In most instances, when the requests related to patient’s own cancer treatment, they were attached to informational updates related to cancer treatment or specific challenges. For example, a patient provided an update on her health status and specifically implored that friends kept praying on her behalf:

Thank you all for the positive thoughts and prayers today...I felt them, even if I didn’t get the results I wanted. Doctors have some additional testing they want to do so they can determine the type of chemo I will receive before surgery. So...more waiting but hopefully tests will come back fine and we can proceed. Keep those prayers coming please.

Patient 22, aged 39 years, diagnosed in 2015

In addition to requests for thoughts and prayers around their own cancer treatment and recovery, cancer patients also requested emotional support for both general cancer– and non–cancer-related issues, including emotional support requests on behalf of others. For example, one of our participants requested emotional support for a friend:

[Friend] just left for his angiogram which he didn’t want to tell anyone about, but I can tell people so I can ask you to please keep him in your thoughts?

atient 14, aged 45 years, diagnosed in 2013

It is important to note that for the women in our study, emotional support exchanges extended beyond both themselves and their cancer to include others in both their Web- and non–Web-based friend networks.

#### Informational Support

Informational support exchange through Facebook was exceptionally limited with only 159 requests for information and 49 instances of unsolicited support–providing posts. In addition, almost none of the 208 informational support exchanges in our 21,291 total coded posts included information about cancer, its treatment, side effects, lifestyle, or diet. In this case, cancer patients on the Web tend to behave much more similar to those outside it in their preference for emotional and resource support and reluctance to exchange information through Facebook.

#### Resource Support

There were two primary types of resource support requests that featured on cancer patients’ Facebook walls. The first were awareness or fundraising campaigns. Though we did not distinguish between the two categories in our coding, this type of awareness and fundraising campaign post might best be termed broadcasting posts. Many of the patients in our sample became very active in raising funds and awareness for both cancer in general, pediatric cancers, or breast cancer subsequent to their cancer diagnosis. Patients would then use their walls as a space to broadcast information on fundraising initiatives to friends and family. For example, a patient campaigned consistently to raise money for childhood cancer and used her Facebook page to raise both awareness and funding for the foundation:

Guess what? If 17 of y’all gave up drinking one green beer or cup of coffee this weekend ($5 only) I would reach $3300 - and be that much closer to helping find a cure for kids cancer. This is your last chance to help support me before I shave my head tomorrow!

Patient 13, aged 41 years, diagnosed in 2011

In addition, several cancer patients sought to raise cancer awareness or share stories of other cancer patients who were either in treatment for cancer or had died following cancer treatment. To that end, many patients used their Facebook pages to request that their friends *spend a minute* visiting a Web page, clicking a link, or reading the story of a fellow cancer patient. As these requests were not always information based, we opted to conceptualize time as a resource and code these requests as resource support requests.

A second category of resource requests corresponded more closely with two common conceptualizations of resource support in social psychology literature. In these cases, patients made direct requests for the resources that they needed to attend to obligations both in and out of the hospital. Some of the materials were things that the patients needed to be more comfortable while in the hospital, such as the patient who requested socks. Other requests centered on resources that could be transmitted over the internet. For example, unable to attend her child’s recital, a patient rallied support through Facebook to obtain photos or videos of the event:

Since I am missing the concert this morning, if anyone takes video’s [sic] or pictures and captures my kiddos could you send them my way, please.

Patient 9, aged 45 years, diagnosed in 2014

These requests were more specific to a patient’s own situation and directed at particular members of her friend network who were local and attending the school concert. As a result, it may make sense for future iterations of the coding schema to distinguish them from general fundraising, awareness raising, and broadcasting posts.

Resource support providing posts were similarly divided. Broadcasting posts were those in which friends reposted a cancer patient’s request for fundraising or other types of support. As mentioned before, this type of support was seen as allowing the cancer patient to access a new network of individuals with requests for support and required time on the part of the support provider. A patient’s fundraiser in support of children with cancer was spread by several of her friends:

We are heading downtown tomorrow night to support our friend Naomi Bleecker Damask while she shaves her head to raise money for kids battling cancer through St. Baldrick's. She is doing this for other kids while in the thick of her own cancer battle, so couldn't we all take 5 minutes out of our busy day to donate just $5 or whatever you can spare to help a child who is battling cancer. Thank you!!!!!!!!!!

Friend, patient 13, aged 41 years, diagnosed in 2011

In addition, there were several instances in which a friend, often a partner, provided an update on the cancer patient’s health status. Although naturally, this would fall under informational support between the patient’s partner and her friends, this was coded as another resource support provision under the broadcasting category in that it required time on the part of the provider and was a service (whether solicited or unsolicited) provided to the patient.

Another complication that arose from the data was how to manage the fact that most of the resources requested by a patient or provided to the patient could not be transmitted over the internet. When the friend of a cancer patient offered to pick the patient’s child up from school and drop the child off at home, this was a clear intention to provide resource support but did not include the transmission of any actual resources. For purposes of this study, we coded any intention to share resource support as a resource support exchange. Proportionally, it is likely that not all of the intentions expressed actually manifested in the transmission of resources, but the intention to transmit resources gives researchers an idea of who is willing and able to provide those resources, and it might be interesting to evaluate the degree to which intention to transmit resources translates into actual transmission of those resources.

#### General Advice

Some posts made by patients were clear requests for engagement but did not include a request for emotional, informational, or resource support. Although infrequent, these posts offered a situation or choice and asked Facebook friends to offer advice on the choice to be made. In an example, a patient asked:

Another house question: double sinks with little counter space or 1 sink with more counter space? I’m wanting the counter space. I only need the sink for two minutes.

Patient 38, aged 33 years, diagnosed in 2016

These were often not cancer-related nor did they pertain to any factual information—rather, solicited the opinions of friends and family connected through Facebook. As there was no preexisting typology available in the support literature to categorize the request, our team opted to create a fourth, *general advice* category. This category subsumed any general solicitations of the opinion of the Facebook network in general.

### Response Metrics

In this phase of the project, we opted to hand code only the 21,291 top-level posts. The approximately 80,000 remaining posts could provide unique insights into the nature and quality of network response to both support requests and provision, and coding schemes for these remaining responses are currently underway. In the meantime, metrics such as the number of likes and comments have been used to assess network response to support requests. However, those metrics were not available, as Facebook only had a *like* button until 2018. This created complications for acknowledging a significant event, observation, or disclosure if the post was characterized by negative valence. In addition, the number of responses had the potential to be complicated by instances in which friends began talking or arguing in the comments section of a specific post. The result of the latter could be a post with multiple comments—possibly interpreted as significant and broad support exchange but included no exchange with the actual person posting. To remedy this, we created an algorithm to count the number of unique commenters. In this way, we could look at the number of comments with respect to that of unique commenters to assess whether a conversation had developed, or the support came from a number of different sources. It is noteworthy that few sources of many comments may not indicate low-quality support if the patient starts a more involved conversation with a friend in the comments section of a post; it may simply indicate that the nature of the support exchanged is likely to be different, and such differences warrant mention and additional attention in analysis.

### Timestamp

In addition to understanding the changes in each patient’s support-seeking behavior following cancer diagnosis, we wanted to look for patterns that were consistent between patients and assess the degree to which support fluctuated over time and with respect to other transitions in health status. To do this, our team used a Unix timestamp for each post and comment. We also used a Unix timestamp for patient’s date of diagnosis and their transition off cancer therapy. This allowed us to superimpose all 30 patients’ 6-month Facebook posting trajectory to view broad-level trends in our sample’s posting behaviors across time to highlight any patterns in fluctuations that might serve as rich points for additional qualitative and quantitative analysis.

### Data

**Figure 1:**
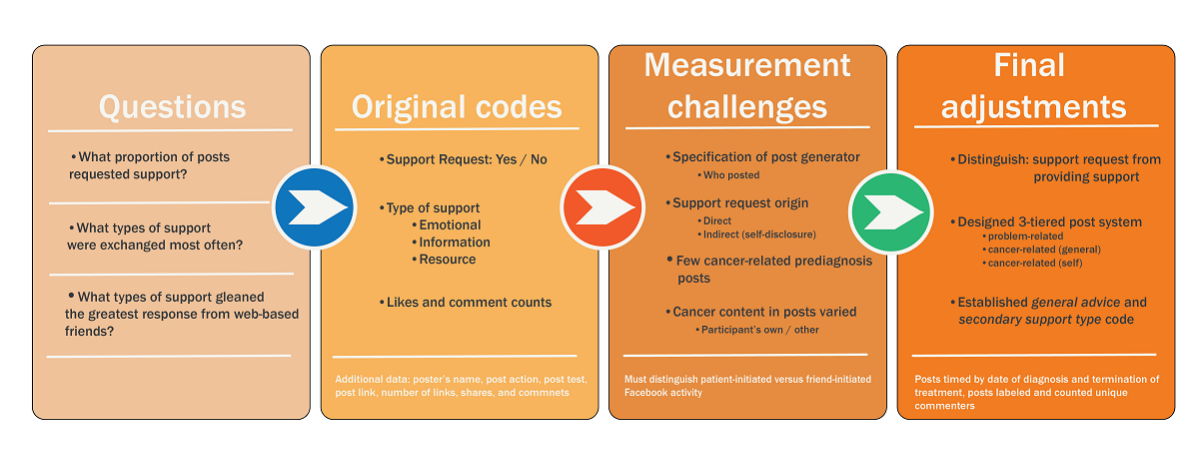
Final coding scheme progression.

**Figure 2:**
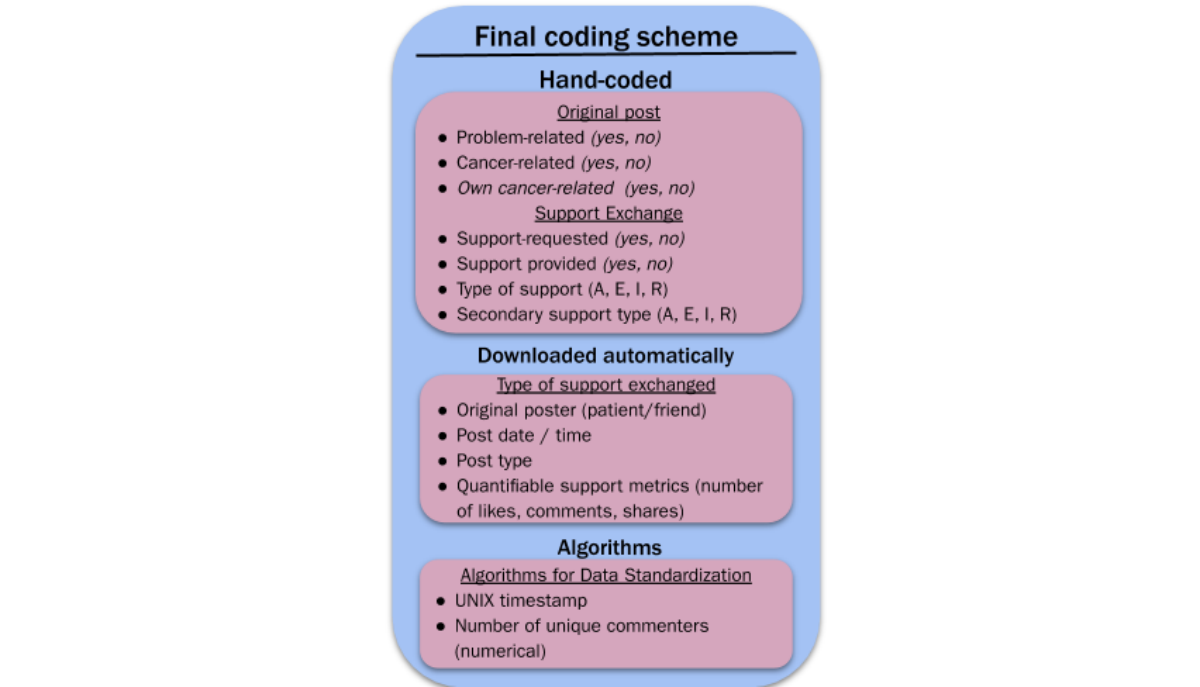
Final coding scheme. I: informational; A: general advice; E: emotional; R: resource.

Figure 1 graphically illustrates how our coding scheme was developed and refined based on observations from the originally downloaded dataset, and Figure 2 shows our final coding scheme. Tables 2 and 4 provide a broad overview of the resulting dataset and include disaggregation by (1) patient, friend, and total posts, (2) proportion of total posts initiated by the patient versus her Facebook friends, (3) post content, including problem-focused, cancer-focused, and patient’s own cancer–focused posts, and finally, (4) proportion of posts that included support exchange broken down by information, advice, resources, and emotional support.

**Table 4:** Summary statistics—posts.

Categories	Friend posts (N=4379), n (%)	Patient posts (N=16,912), n (%)	Total posts (N=21,291), n (%)
Problem-related	664 (15.23)	2006 (11.89)	2670 (12.57)
Cancer-related	920 (21.09)	1889 (11.19)	2809 (13.22)
Patient’s cancer	720 (16.51)	1358 (8.05)	2078 (9.78)

From the tables, we see limited requests for support and a mismatch between the types of support being requested and that being provided by friends. Although patient’s own posts account for the large majority of overall posts, the overall proportion of patients’ posts that include a request for support is only around 3%, with self-disclosures at around 8%. In addition, over half of patient requests are for resource support (59%), whereas only 27% request emotional support. By contrast, friends’ posts were twice as likely to bring up the patient’s own cancer and over 9 times as likely to include support. However, despite patients requesting a preponderance of support requests, the overwhelming majority of support provided (80%) was emotional support, with only around 19% offering resource support.

## Discussion

### Principal Findings

We set out to measure support exchange around two critical transitions in cancer treatment: diagnosis and transition off cancer therapy. Our preliminary review of the more than 100,000 lines of data collected from 30 breast cancer patients’ Facebook activity surrounding each transition revealed more questions than answers. Were we interested in support around any and all issues or simply around breast cancer? Will a support request always include a statement of need or want, or can Facebook users request support by simply disclosing information about their situation? How do we count unsolicited posts from friends that contain supportive, if limitedly substantive, statements such as, *you rock*? And perhaps even more muddy, how do we manage situations where participants are tagged in someone else’s post or where husbands post updates from their wives’ Facebook account? These issues are made all the more difficult by the fact that rationale (and often empirical precedent) exists to justify either decision.

Answering these broader questions began with a survey of the literature and a guiding philosophy that favored theoretically grounded divisions. We began with the data that were scraped automatically from our browser extension and then created variables that would allow us to explore theoretical concepts in support exchange. These included dichotomous variables for support exchange and valence and a categorical variable for type of support exchanged. Finer distinctions emerged iteratively in coding, as our team encountered status updates and wall posts that could not be fit to the original coding scheme. A total of 8 themes emerged in two categories, specifically data comparability and operationalization of support. Comparability refers to grouping similar components. In other words, if we are interested in social support exchanges, it is important to distinguish between posts created by patients and those created by friends, that predate cancer diagnosis, and that do and do not request support. Other distinctions resulted from a difficulty in operationalizing support and resulted in adapting categories and definitions to suit the nature of the data, from counting requests to read as a request for friends’ time (resource support) to creating a new category for general advice to cover posts requesting opinions on hair styles or dresses.

The resulting dataset provides unique insights into the nature and quality of support exchanges via Facebook. Though derived from a smaller sample of breast cancer patients, the database contains 21,291 individual posts from Facebook users and provides unique insights into the utility and responsiveness of social support exchanges via Facebook. Broad-level data categorizations indicate that Facebook may be effective in allowing patients to fulfill social obligations and broadcast general needs but provides social support that is very general and does not respond to any specific or cancer-related support need. To illustrate, despite 16,912 status updates, only 3% of posts included a direct request for support, and the majority of those support requests were for resource support. By contrast, friends’ posts accounted for only 20% of overall posts but were twice as likely to include reference to the participant’s own cancer and 9 times as likely to include support. However, the overwhelming majority of unsolicited support provided by friends was emotional, with less than one-fifth offering resources—time or services.

Given the importance of social media in social interaction, support exchange, and health promotion, our findings offer a timely contribution to methods of evaluating support exchange in social media environments. Current trends in social media research and Web-based peer exchange show important avenues for future insights on the value of social media to overcome communication barriers normally associated with in-person or more formal and potentially prescriptive modes of social support exchange, but such research has been hampered by a need for consistent operationalizations of online social support that cuts across studies. Where this study offers insight beyond existing comparisons of social support exchange with Web-based economies of information exchange [54], is the way this coding schema permits a more compelling analysis of support exchange as a type of *peer community*, where members act proactively to buffer stress effects associated with negative health experiences.

Reviews of qualitative or mixed-methods approaches to Web-based data analysis point to trends in research using counts and content and thematic analysis; however, few studies show researchers applying strategic approaches to answer specific research questions using Facebook or other social media data [55]. This study addressed two of those issues by providing a systematic coding schema that allowed a characterization of social exchange behaviors to suggest that Facebook use for social support extended beyond information provision and instrumental support to include socioemotional support. In addition to providing clarity on social media behaviors for cancer patients, our paper outlines a clear method and design for organizing and assessing unwieldy social media data in useful ways. Such an approach addresses a gap in the field emphasized by recent review data showing that better research designs and methods are needed for examining the effectiveness of social media platforms for health benefit [3].

### Conclusions

Social media data are unwieldy and not always conducive to neat categorizations. Designing a data analysis approach a priori for organizing and systematizing Facebook data allows us to explore the functionality of Facebook as a platform for the exchange of social support. In doing so, this paper provides insights into the different forms that informational, resource, and emotional support may take in Web-based social environments such as Facebook and challenges researchers may face in measuring those constructs and how to respond to such challenges to create broader continuity in evaluation and measurement of computer-mediated support exchange.
